# Cytochrome P450 1A2 Metabolizes 17β-Estradiol to Suppress Hepatocellular Carcinoma

**DOI:** 10.1371/journal.pone.0153863

**Published:** 2016-04-19

**Authors:** Jianwai Ren, George G. Chen, Yi Liu, Xianwei Su, Baoguang Hu, Billy C. S. Leung, Y. Wang, Rocky L. K. Ho, Shengli Yang, Gang Lu, C. G. Lee, Paul B. S. Lai

**Affiliations:** 1 Department of Surgery, Faculty of Medicine, The Chinese University of Hong Kong; New Territories, Hong Kong, China; 2 CUHK Shenzhen Research Institute (SZRI), Shenzhen, 518057, China; 3 Guangdong Key Laboratory for Research and Development of Natural Drugs, Guangdong Medical University, Zhanjiang, Guangdong, China; 4 National Cancer Centre, Division of Medical Sciences, Singapore, Singapore; National University of Singapore, SINGAPORE

## Abstract

Hepatocellular carcinoma (HCC) occurs more frequently in men than in women. It is commonly agreed that estrogen plays important roles in suppressing HCC development, however, the underlying mechanism remains largely unknown. Since estrogen is mainly metabolized in liver and its metabolites affect cell proliferation, we sought to investigate if the liver-specific cytochrome P450 1A2 (CYP1A2) mediated the inhibitory effect of estrogen on HCC. In this study, the expression of estrogen-metabolizing enzyme CYP1A2 was determined in HCC tissues and cell lines. Cell proliferation and apoptosis were assessed in cells with or without *CYP1A2* overexpression. The levels of 17β-estradiol (E2) and its metabolite 2-methoxyestradiol (2-ME) were determined. A xenograft tumor model in mice was established to confirm the findings. It was found that *CYP1A2* expression was greatly repressed in HCC. E2 suppressed HCC cell proliferation and xenograft tumor development by inducing apoptosis. The inhibitory effect was significantly enhanced in cells with *CYP1A2* overexpression, which effectively conversed E2 to the cytotoxic 2-ME. E2 in combination with sorafenib showed an additive effect on HCC. The anti-HCC effect of E2 was not associated with estrogen receptors ERα and ERβ as well as tumor suppressor P53 but enhanced by the approved anti-HCC drug sorafenib. In addition, HDAC inhibitors greatly induced *CYP1A2* promoter activities in cancer cells, especially liver cancer cells, but not in non-tumorigenic cells. Collectively, CYP1A2 metabolizes E2 to generate the potent anti-tumor agent 2-ME in HCC. The reduction of CYP1A2 significantly disrupts this metabolic pathway, contributing the progression and growth of HCC and the gender disparity of this malignancy.

## Introduction

Hepatocellular carcinoma (HCC) is one of the most common and fatal malignancies worldwide. Although it has been well documented that the incidence of HCC is higher in males than in females [1], the underlying mechanism still remains largely unknown. Various factors have been proposed to contribute to the gender difference of HCC incidence. Genetic alterations of chromosome Y and chromosome X have been frequently observed in HCC patients [2,3], indicating the genes that are located on sex chromosomes may play roles in HCC development. Unhealthy lifestyles such as smoking and alcohol consumption that are more prevalent in men than in women are also speculated to be one of the reasons for the gender disparity [4]. But most of all, extensive investigations have demonstrated that sex steroid hormones may play a dominant role in causing the gender disparity of HCC development [5,6].

Both of androgen and estrogen have been reported to function in HCC development [6]. However, the stimulating effect of androgen awaits further verification due to the fact that the androgen effect is mainly inferred from the study on androgen receptor [7,8], whereas the preventive or inhibitory effect of estrogen has been epidemiologically demonstrated by solid cohort studies showing higher HCC incidence rate after menopause [9–11], and directly confirmed in animal models showing the decrease of HCC incidence or HCC metastasis in estrogen-treated individuals [12]. In addition, experimental data appear to be consistent in supporting the epidemiological and animal findings, as estrogen can inhibit HCC by regulating several signalling pathways including the induction of apoptosis in HCC cells, inactivation of the liver macrophages, downregulation of proinflammatory cytokines, suppression of NF-κB and targeting IL-6 and STAT3 [13].

Studies have shown that the effects of estrogen on HCC were mediated by estrogen receptors, ERα and ERβ, including their splicing variants. However, the involvement of these receptors in hepatocarcinogenesis remains inconsistent since both anti-HCC and pro-HCC effects of estrogen receptors have been reported. For example, Xu et al and Shi et al showed that ERα is inhibitory on HCC progression by inactivating NF-κB and STAT3 [14,15], whereas it was also demonstrated that estrogen receptors might promote HCC development by downregulating peroxisome proliferator-activated receptor γ (PPARγ) or interfering with Wnt pathway [16,17]. Although the function of estrogen is typically executed by binding to one or more of its receptors, increasing evidences have shown that estrogen may also function via its interaction with other molecules or/and indirectly through its metabolic products, both of which can be independent of its receptors. 17β-estradiol (E2), the most potent form of estrogen, is metabolized by several cytochrome P450 enzymes, some of which may have tissue-specific distribution. Liver, the major organ for E2 metabolism [18], metabolizes E2 primarily by cytochrome P450 1A2 (CYP1A2), and to a lesser degree by CYP3A4 [19].

Both of CYP1A2 and CYP3A4 convert E2 to 2-hydroxyestradiol which is further methoxylated by catechol-O-methyltransferase or (COMT) to generate 2-methoxyestradiol (2-ME). Increasing experimental data have demonstrated that 2-ME is a potent anti-cancer agent *in vitro* and *in vivo*. 2-ME can inhibit HCC via multi-channels including suppression of angiogenesis, cell cycle arrest, and induction of apoptosis [20,21]. Despite the fact that estrogen metabolite 2-ME is a known anti-HCC agent and that CYP1A2, CYP3A4 and COMT are the key enzymes responsible for the production of 2-ME, little is known about their E2 metabolism in HCC. We thus hypothesized that HCC is associated with defect in one or more of these three enzymes, and that the defect may cause insufficient metabolism of E2 and the under-production of 2-ME, which damages the self-protection system in liver and facilitates the development, progression and growth of HCC. If this concept is proved to be the case, it can offer a new explanation for the gender disparity of HCC incidence.

In this report, we have demonstrated that the expression of *CYP1A2* was significantly reduced in HCC. Overexpression of *CYP1A2* in HCC cells reduced the content of E2 but increased the level of 2-ME. HCC cells treated with E2 or 2-ME were much less proliferative and with increase of apoptotic cells. The suppressive effect of E2 was enhanced by overexpression of *CYP1A2* but not by *CYP3A4* or *COMT* overexpression. Xenograft tumors derived from *CYP1A2*-overexpressing HCC cells developed much slower. Our results have suggested for the first time that E2 metabolism may play important roles in suppressing the proliferation and growth of HCC. The findings support the protective role of E2 in hepatocarcinogenesis, which may contribute to the lower incidence of HCC in females.

## Materials and Methods

The human study was approved by the joint Chinese University of Hong Kong—New Territories East Cluster Clinical Research Ethics Committee. The participants provided their written informed consent to participate in this study. The animal experiments were approved by the Chinese University of Hong Kong Animal Experimentation Ethics Committee.

### Tissues, cell culture, and chemical reagents

All HCC tissue specimens accompanied by clinical information (S1 Table) were collected. Human liver cancer cell lines Hep3B, C3A, Huh7, PCL/PRF/5were purchased from American Type Culture Collection (ATCC, Manassas, VA) and cultured in 10% FCS supplemented DMEM in a 5% CO2 atmosphere at 37°C. All chemicals including β-estradiol (E2), and 2-ME were purchased from Sigma-Aldrich (St. Louis, MO) unless otherwise stated. Stock solutions were prepared by dissolving the chemicals in DMSO to reach a 50mM concentration. Hence in drug treatment DMSO was used as vehicle control.

### Generation of plasmid constructs and stable cell lines

*ERα*, *ERβ*, *GPR30*, *CYP1A2*, *CYP3A4* and *COMT* were cloned for functional studies. Briefly, the coding regions of these genes were amplified via RT-PCR from total RNA extracted from human liver tissues and cloned into HindIII/NotI site of pcDNA3.1(+) vector (Invitrogen, Carlsbad, CA). The sequences were verified and the activities of the plasmid-encoded proteins were confirmed via respective assays. To evaluate the activities of ERα and ERβ, the estrogen-responsive luciferase reporter plasmid was co-transfected and the elevated reporter gene expression induced by subsequent E2 treatment was analyzed with luciferase assay system (Promega, Madison, WI) (S2 Table). The activity of GPR30 was examined based on the fact that estrogen induces apoptosis in GPR30 overexpressing cells [22,23] (S1 Fig). The activities of CYP1A2, CYP3A4 and COMT were examined via cell-based analysis with respective P450-Glo^™^ Assays (Promega, Madison, WI) (S3 Table). CYP1A2-stably expressing Hep3B cell line was established via G418 selection and the positive clones were confirmed by CYP1A2 enzyme activity assay. Nine clones of each of control and CYP1A2 stable cells were respectively combined in one culture and expanded for subsequent studies.

### Proliferation and apoptosis assays

Cell proliferation was evaluated with MTT method as previously described [14,24,25]. Each assay was performed in four replicates. For apoptosis analysis, the cells were trypsinized and harvested by centrifugation and then fixed in cold 70% ethanol. Afterwards the cells were stained with Propidium Iodide (PI)/RNase Staining Solution (Cell Signaling Technology, Danvers, MA) and subjected to FACSCalibur flow cytometer (BD Biosciences, Franklin Lakes, NJ). Apoptotic cells were identified as the sub-G1 peak of the DNA content profiles.

### Scratch-wound healing assay

Control and CYP1A2 stable Hep3B cells were grown to confluence and a 2mm-thin wound gap was generated by scratching with a rubber policeman. The cells were then grown in medium supplemented with or without 1μM E2 for 10 days before microscopic images were taken.

### RT-qPCR

Total RNA was extracted from tissues or cells using TRIzol reagent (Invitrogen) according to the manufacturer’s protocol. RT was performed using 0.5 μg of RNA, oligo (dT)20 primer and SuperScript III kit (Invitrogen) in 10 μl reaction. For qPCR, 0.1 μl of the reverse transcription product was used with respective primers (S4 Table) in 10 μl reaction with SYBR^®^ Green qPCR Master Mixes (Invitrogen). The analysis was done using the ABI7900HT Real Time PCR system (Applied Biosystems). Four replicate wells were read for each sample. In examining expression level of a gene, the qPCR reactions for the target gene as well as the reference gene, β-Actin, were simultaneously run together in one single 384-well plate to prevent the inter-place variation. The relative expression level in each sample was quantified against the reference gene.

### ELISA and Western blot

E2 and 2-ME EIA kits (Cayman Chemical, Ann Arbor, MI) were used to measure the cellular contents of E2 and 2-ME. Hep3B was transfected with CYP1A2 and grown in E2 supplemented medium for 24 hours. Afterwards the cells were harvested and sonicated in PBS. The cell lysate was used to measure E2 and 2-ME levels with respective EIA kits following the manufacturer’s instructions. Each sample was measured in triplicate, and the results were normalized to the amount of protein in each sample.

20–30 μg of protein extracted from HCC tissues was used for Western blotting as described previously [26]. All the antibodies used in the study were purchased from Santa Cruz Biotechnology (Santa Cruz, CA).

### Xenograft tumor model

All animal experiments were carried out in accordance with institute guidelines on animal experimentation. 4-week old male BALB/c nude mice were supplied by CUHK animal center. Three independent experiments were conducted. In each experiment, 4 nude mice were used. The mice were maintained on a 12 h/12 h light/dark cycle and were provided with standard chow and water access ad libitum. Xenograft tumor models were generated by injecting 1x10^7^ control or CYP1A2-stable Hep3B cells subcutaneously in the dorsal sides near right hind limbs of the nude mice. Two days later, the mice were injected with E2 (50 μg/kg) or vehicle control (0.02% DMSO in PBS) intraperitoneally once-daily for 10 days. Afterwards the mice were maintained as above mentioned and the growth of the tumors was monitored every day. When one dimension of the largest tumor reached 1cm (about 4 weeks), all the mice in each experiment were euthanized by quick cervical dislocation and the tumors were collected and weighed. All the mice were still active before euthanasia. The results in three independent experiments were combined for statistical analysis

### CYP1A2 promoter assay

The 5kb promoter region upstream of the *CYP1A2* transcription start site (TSS) was cloned in pGL3 Luciferase Reporter Vector (Promega, Madison, WI) and the construct was verified by sequencing. In promoter assay, the cells were transfected and then re-seeded at 1:3 split. Afterwards the cells were grown in the drug-supplemented medium for 24 hours and then lysed for Luciferase (Luc) activity assay. The Luc reading was normalised with protein concentration. Concentrations of the drugs were determined as the minimum level that caused obvious difference in cell proliferation assays.

### Statistics

Statistical significance was determined by two-tailed Student’s t-test or one-way ANOVA followed by the Student’s t-test.

## Results

### E2 and 2-ME suppress the proliferation and induce apoptosis in HCC cells

**Fig 1:**
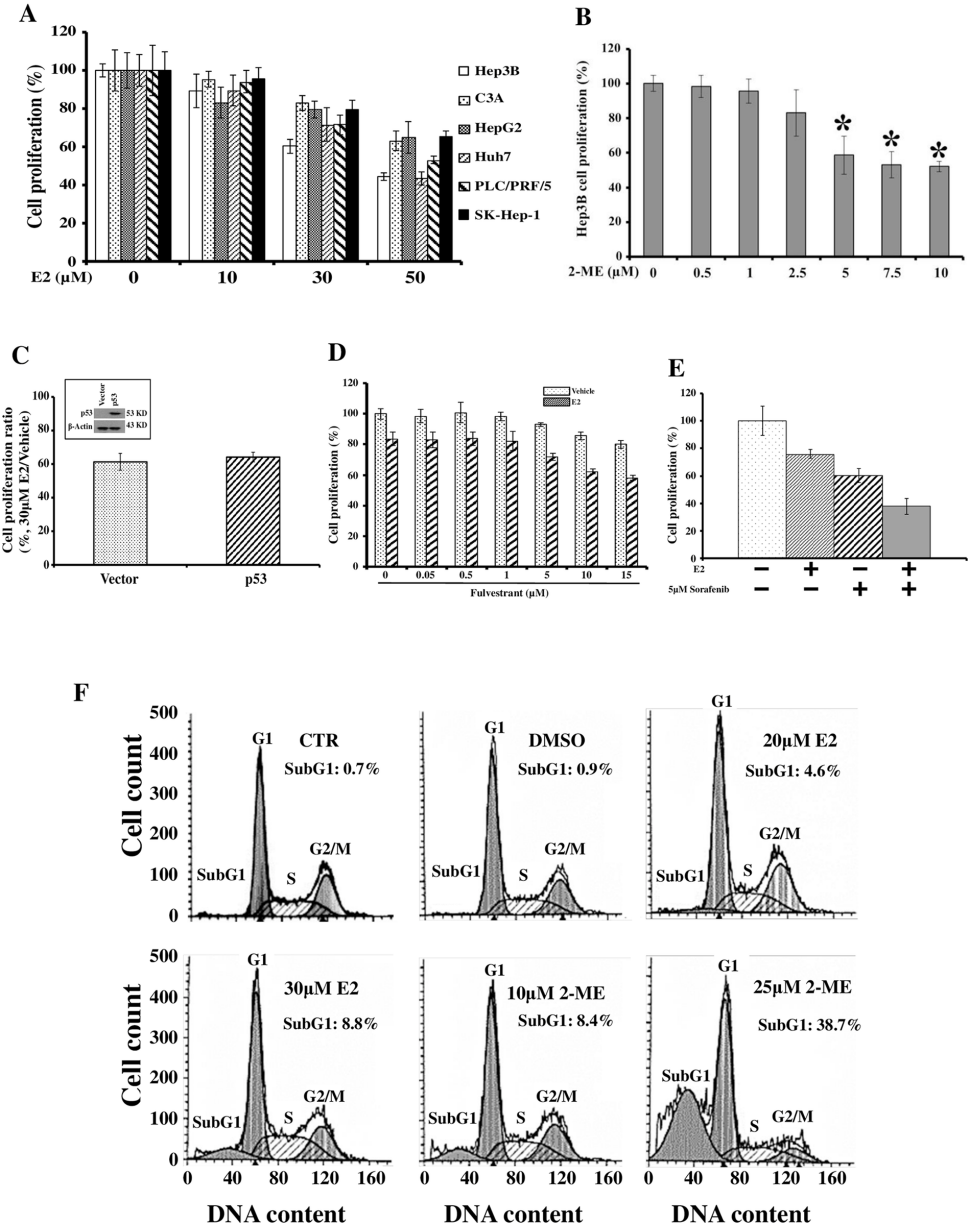
Estrogen suppresses proliferation of liver cancer cells. In **A–E** HCC cells were seeded at 5x10^3^ cells/per well of 96-well plates and grown for 24 hours. The cells were treated as indicated and analyzed for proliferation rate by MTT. Each column represents mean±s.d. of data obtained from four replicate wells. Consistent results were obtained in three independent experiments. **(A)** E2 inhibited proliferation of HCC cells. The cells were treated with E2 of the indicated concentrations for 48 hours before MTT assay. The proliferation rate of individual cell line is expressed as the value of treated cells versus that of the untreated cells (= 100%). **(B)** 2-ME inhibited Hep3B cell growth in a dose-dependent manner. Hep3B was treated with 2-ME of the indicated concentrations for 48 hours before MTT assay. The proliferation rate is expressed as the value of treated Hep3B cells versus that of the untreated Hep3B cells (= 100%). **(C)** Inhibitory effect of E2 is not influenced by P53. Hep3B cells were transfected with wt p53 DNA or empty vector control. Inset shows the expression of *p53* in the transfected cells. The proliferation rate is expressed as the value of treated cells versus that of the untreated cells of the same transfection (= 100%). **(D)** The Hep3B cells were treated with fulvestrant of indicated concentrations supplemented with or without 30μM E2 for 48 hours before MTT assay. E2 inhibited the HCC cell proliferation in a way basically independent of the increasing doses of fulvestrant. **(E)** E2 additively enhanced the effect of sorafenib in suppressing the growth of Hep3B cells. **(F)** E2 and 2-ME induced apoptosis in Hep3B cells. The cells were treated with E2 or ME2 for 48 hours before being harvested for PI staining. Apoptotic cells were identified as the sub-G1 peak of the DNA content profiles.

To examine the effects of E2 and 2-ME on the proliferation of HCC cells, cells were treated with different doses of E2 “Fig 1A” or 2-ME “Fig 1B” for 48 h. The treatment time was determined by carrying out several optimization experiments in which we found that both of short treatment time and high cell confluence attenuated the inhibitory effects of E2 and 2-ME, indicating that only dividing cells responded to the reagents. At the end of the treatment, cell proliferation was measured by MTT assay. Both E2 and 2-ME were able to inhibit HCC cell proliferation. However different cell lines responded differently to E2 treatment. For example, 30μM E2 could lead to 40% inhibition of Hep3B cell proliferation whereas only 20% in HepG2 cells. It was noted that the suppressive effect of E2 only appeared at high doses of E2 though as low as 10nM E2 was able to activate the estrogen receptors (S2 Table, S1 Fig).

**Fig 2:**
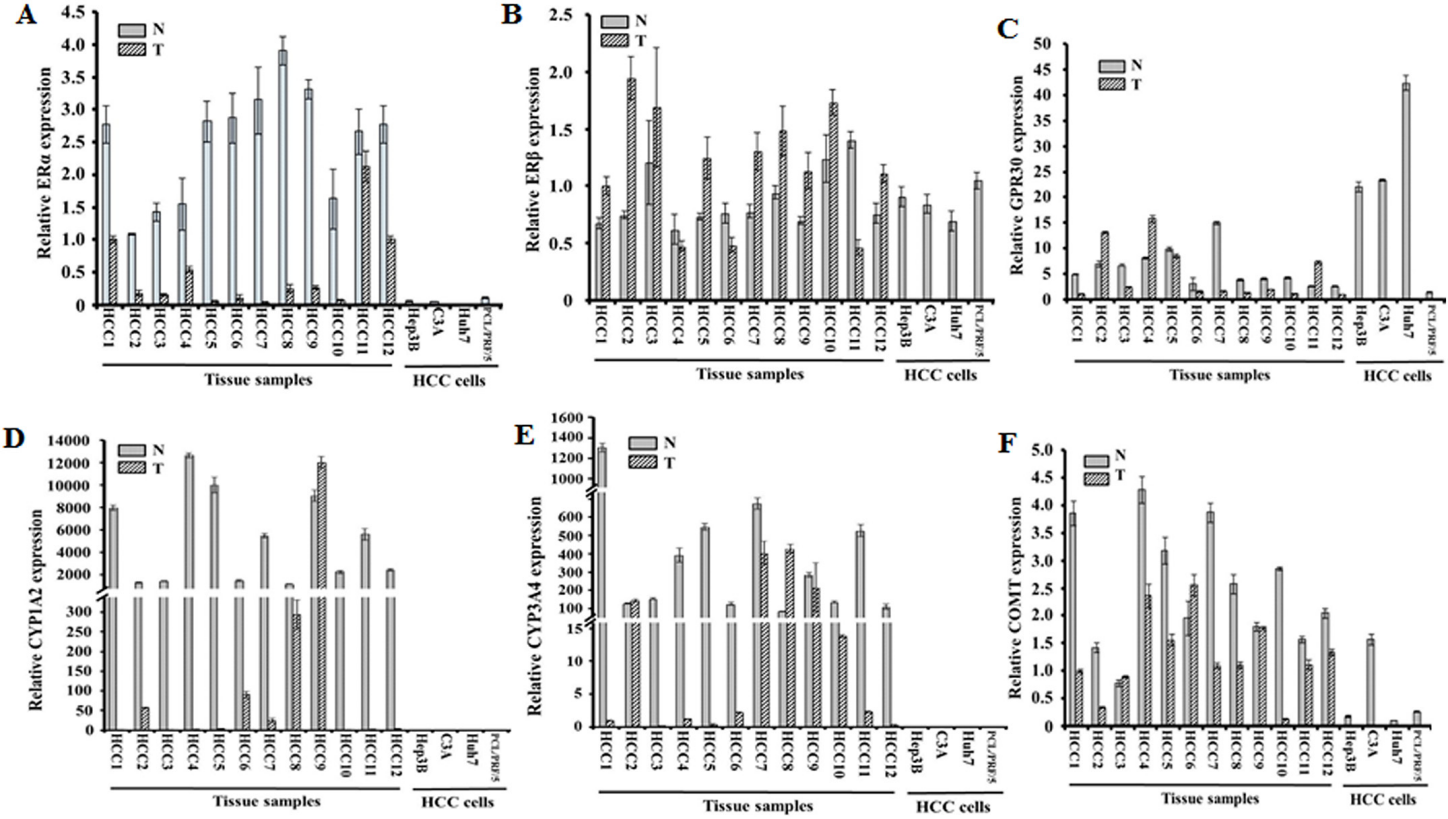
Expression of *ERα*, *ERβ*, *GPR30*, *CYP1A2*, *CYP3A4* and *COMT* in HCC. Total RNA was extracted from 12 pairs of human HCC samples (tumorous (T) and adjacent non-tumorous (N) tissues) and human HCC cell lines. Among the tissue samples, HCC1, 3, 5, 7 and 10 were obtained from female patients, the rest were obtained from male patients (S1 Table). The mRNA levels of *ERα* (**A**), *ERβ* (**B**), *GPR30* (**C**), *CYP1A2* (**D**), *CYP3A4* (**E**) and *COMT* (**F**) were analyzed by RT-qPCR. The expression levels were normalized with β-Actin mRNA levels. The data are expressed as fold changes relative to the expression level in the tumor of HCC1. Each column represents the mean ± s.d. of four technical replicates.

The obvious difference between Hep3B and HepG2 is that the former is a *p53*-defecient cell line where the latter contains wide-type *p53*. In order to check whether p53 is a factor causing the different responses to E2 in various cell lines, we transfected Hep3B with wide-type p53, followed by E2 treatment. Fig 2C shows that p53 did not affect the estrogen inhibitory effect on Hep3B growth. Our finding further suggested that the inhibitory effect of E2 might not be related to ERα because the degree of E2-mediated inhibition relative to non-E2 treatment remained almost the same along the increasing dose of fulvestrant “Fig 1D”, which may downregulate and degrade ERα [27]. Sorafenib is the only approved systemic anti-HCC for patients at advanced stages and current studies have suggested that sorafenib be used together with other anti-tumor agents to increase the efficacy [28]. It will thus be interesting to find out whether E2 can be a potential agent to be used in combination with sorafenib. To this end, we found that E2 could significantly enhance the effectiveness of sorafenib, compared with either agent alone “Fig 1E”. Both E2 and 2-ME were found to induce apoptosis in HCC cells, which was reflected by significant increase of cell population in sub-G1 “Fig 1F”. It was noted that the most significant increase of Sub-G1 population was caused by 10μM 2-ME treatment.

### Expression of ERs, CYP1A2, CYP3A4 and COMT in HCC

To explore the mechanism underlying the inhibitory effect of E2, the expression levels of genes related to estrogen function and metabolism in HCC tissues and HCC cell lines were exarmined. These genes include *ERα*, *ERβ*, *GPR30*, *CYP1A2*, *CYP3A4* and *COMT*. It was found that expression of ERα mRNA was down-regulated by 3 to 20 folds in almost all of the HCC tumors compared to the corresponding non-tumorous tissues “Fig 2A”. In addition, the expression levels of ERα were very low in all HCC cell lines compared with that of HCC samples. There was no difference in ERβ expression mRNA between tumors and adjacent normal tissues of the HCC patients “Fig 2B”. The levels of ERβ mRNA in HCC cell lines were similar to that in HCC tissue samples. *GPR30* was differentially expressed in many HCC samples but its overall level was not significantly different between tumors and non-tumorous samples “Fig 2C”. However, it was noted that *GPR30* was highly expressed in most HCC cell lines compared with that of HCC samples. The expression of CYP1A2 mRNA was greatly suppressed in 11 of the 12 HCC tumors compared with that in the non-tumorous samples. In most cases the fold-changes were more than 1000 folds “Fig 2D”. A similar result was found in CYP3A4 mRNA but the downregulation was not as great as that of CYP1A2 mRNA “Fig 2E”. In addition, the levels of CYP1A2 and CYP3A4 mRNA in all HCC cell lines tested were extremely low compared with that of HCC samples. The expression of COMT mRNA varied in HCC. The mRNA levels were increased in 3 out 12 HCC tissue samples but were decreased in the rest samples compared with non-tumorous samples “Fig 2F”. Notably, no gender-specific pattern has been observed in the expression profiles of the estrogen-related genes.

### CYP1A2 facilitates E2 metabolism and enhances E2-mediated suppression

**Fig 3:**
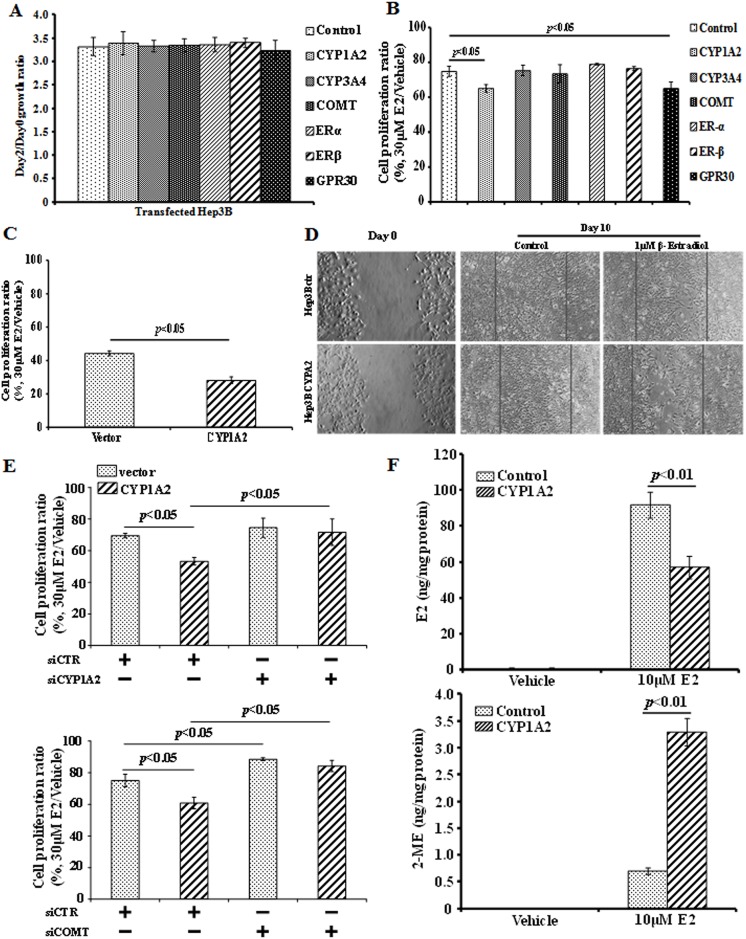
Effects of gene overexpression on E2-mediated inhibition of cell proliferation. **(A)** Growth rate of the transfected cells. Hep3B cells were transfected with vector control or *ERα*, *ERβ*, *GPR30*, *CYP1A2*, *CYP3A4*, *COMT* plasmid DNAs, respectively, and grown for 24 hours. Then the cells were seeded at 5x10^3^ cells/per well of 96-well plates and grown for 24 hours, at which time MTT was performed with one set of the cells (Day0). MTT for another set of the cells was performed 48 hours later (Day2). The growth ratios of the cells are expressed as the value of Day2 cells versus that of the Day0 cells of the same transfection (= 100%). **(B)** Hep3B cells were transfected and reseeded as mentioned in (A). Afterwards the cells were treated as indicated for 48 hours and analyzed for proliferation rate by MTT. The proliferation rate is expressed as the value of treated cells versus that of the untreated cells of same transfection (= 100%). Each column represents mean±s.d. of data obtained from four replicate wells. Consistent results were obtained in three independent experiments. **(C)** CYP1A2 mediates E2-inhibition of cell proliferation in 293T cells. The proliferation rate is expressed as the value of treated cells versus that of the untreated cells of same transfection (= 100%) Consistent results were obtained in three independent experiments. **(D)** CYP1A2 stable Hep3B cells migrated slowly under E2 treatment. Hep3B stable cells overexpressing CYP1A2 or empty vector were grown to confluence and a 2mm-thin wound gap was generated by scratching with a rubber policeman (Day0). The cells were then grown in medium supplemented with or without 1 μM E2 for 10 days (Day10) before microscopic images were taken. The lines indicate the wound edges. Consistent results were obtained in three independent experiments. (**E**) Hep3B cells were co-transfected with siRNA against *CYP1A2* (siCYP1A2) or *COMT* (siCOMT) and *CYP1A2*-expressing plasmids and grown for 24 hours. Then the cells were seeded at 5x10^3^ cells/per well of 96-well plates and grown for 24 hours. Afterwards the cells were treated as indicated for 48 hours and analyzed for proliferation rate by MTT. The proliferation rate is expressed as the value of treated cells versus that of the untreated cells of same transfection (= 100%). Each column represents mean±s.d. of data obtained from four replicate wells. Consistent results were obtained in three independent experiments. **(E)** Hep3B cells were transfected with *CYP1A2* and grown for 24 hours. Then the cells were grown in 10μM E2 supplemented medium for another 24 hours. Afterwards the cells were harvested and lysed in PBS by sonication. Concentrations of E2 and 2-ME were measured with respective EIA kits mentioned in Materials and Methods. The values represent the relative concentrations normalized against the protein concentration in the same sample. Each column represents mean±s.d. of data obtained from three independent assays.

To further elucidate how E2 exerts its suppressive effect on HCC cells, the cells were transfected *ERα*, *ERβ*, *GPR30*, *CYP1A2*, *CYP3A4*, or *COMT* DNA and overexpression of these genes alone did not significantly influence HCC cell proliferation “Fig 3A”. The inhibitory effect of E2 was significantly enhanced in *CYP1A2*- and *GPR30*-overexpressing cells but not in *CYP3A4*, *COMT*, *ERα* or *ERβ*-overexpressing cells “Fig 3B”, indicating the possible involvement of CYP1A2 and GPR30 in the E2-induced inhibition. Considering the liver-specific expression of *CYP1A2* and the fact that the levels of *CYP1A2* but not *GPR30* were significantly reduced in HCC “Fig 2C and 2D”, we found it more interesting to further investigate the functions of CYP1A2 rather than that of GPR30 in mediating the E2-induced inhibition. In addition, the consistent result regarding the effect of CYP1A2 was obtained with 293T cells, excluding the possibility that above CYP1A2 effect was cell line specific. Moreover, due to higher transfection efficiency in 293T cells, a more robust effect of CYP1A2 was observed “Fig 3C”.

In addition to the inhibition of HCC cell proliferation, the overexpression of *CYP1A2* in the presence of E2 could also markedly prevent the aggressive migration of HCC cells as evidenced by the result of wound healing assay (Fig 3D, S2 Fig). Interestingly, when the *CYP1A2* and *COMT* expression was suppressed by siRNA in Hep3B cells, the inhibitory effect of E2 was significantly attenuated, even in the *CYP1A2* overexpressing cells, indicating CYP1A2/COMT metabolic pathway plays an important role in mediating the inhibitory effect of E2 “Fig 3E”. Importantly, in the E2-supplemented medium, the content of E2 in *CYP1A2*-overexpressing cells was significantly lower than that in empty-vector transfected cells “Fig 3F upper panel”. In contrast, the content of E2 metabolite 2-ME was obviously elevated in *CYP1A2*-overexpressing cells “Fig 3F lower panel”. These findings suggest that the overexpression of *CYP1A2* could result in the higher rate of E2 metabolism.

**Fig 4:**
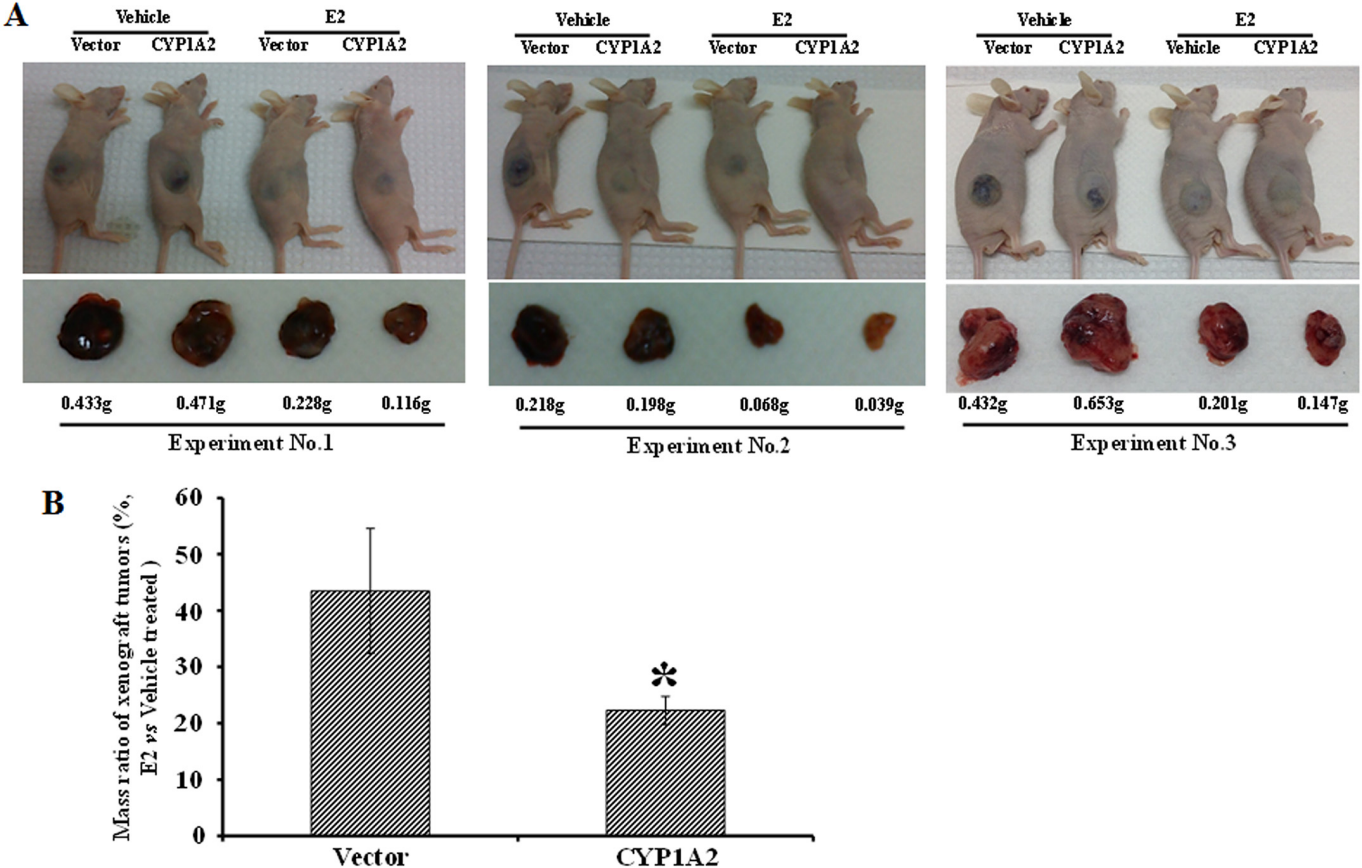
CYP1A2 enhanced the inhibitory effect of E2 on xenograft tumors. Empty vector control and *CYP1A2*-overexpressing Hep3B stable cells were generated and nine clones of each cell line were combined in culture and used *in vivo* animal experiments. 1 x 10^7^ Hep3B cells with *CYP1A2* or empty vector were subcutaneously injected into 4-week old male BALB/c nude mice. Two days later, the mice were intraperitoneally injected with E2 (50 μg/kg) or vehicle control (0.02% DMSO in PBS) once daily for 10 days. Afterwards the mice were maintained for about four weeks before the tumors were collected for analysis. Weights of the tumors were indicated under the tumor images. Three independent xenograft assays were performed. **(A)** Xenograft tumor-carrying mice and the tumors in each experiment. **(B)** E2 suppressed the growth of *CYP1A2*-overexpressing tumors more potently. Mass ratio is expressed as the value of the weight of E2-treated tumors versus the vehicle-treated tumors of each stable cell line (vector or *CYP1A2*). Each column represents mean±s.d. of data obtained from the three independent xenograft assays. *p<0.05 when compared to vector stable cells.

Having demonstrated that CYP1A2 may metabolite E2 to inhibit the proliferation of cultured HCC cells, an important question to ask is whether this is also the case *in vivo*. To answer this question, we established xenograft tumors in mice, in which Hep3B cells with stable *CYP1A2* overexpression were subcutaneously inoculated. We found that stable cells with either empty vectors or *CYP1A2* overexpression could form xenograft tumors at the same rates under normal conditions “vehicle groups, Fig 4A”. E2 treatment led to the reduction in the mass of both cases “E2 groups, Fig 4A”, however E2-mediated reduction of *CYP1A2*-oeverexpressing tumor was more robust than that of the tumor without *CYP1A2* overexpression “Fig 4B, 45% *vs* 22%, *p*<0.05”.

### CYP1A2 expression is up-regulated by HDAC inhibitors

**Fig 5:**
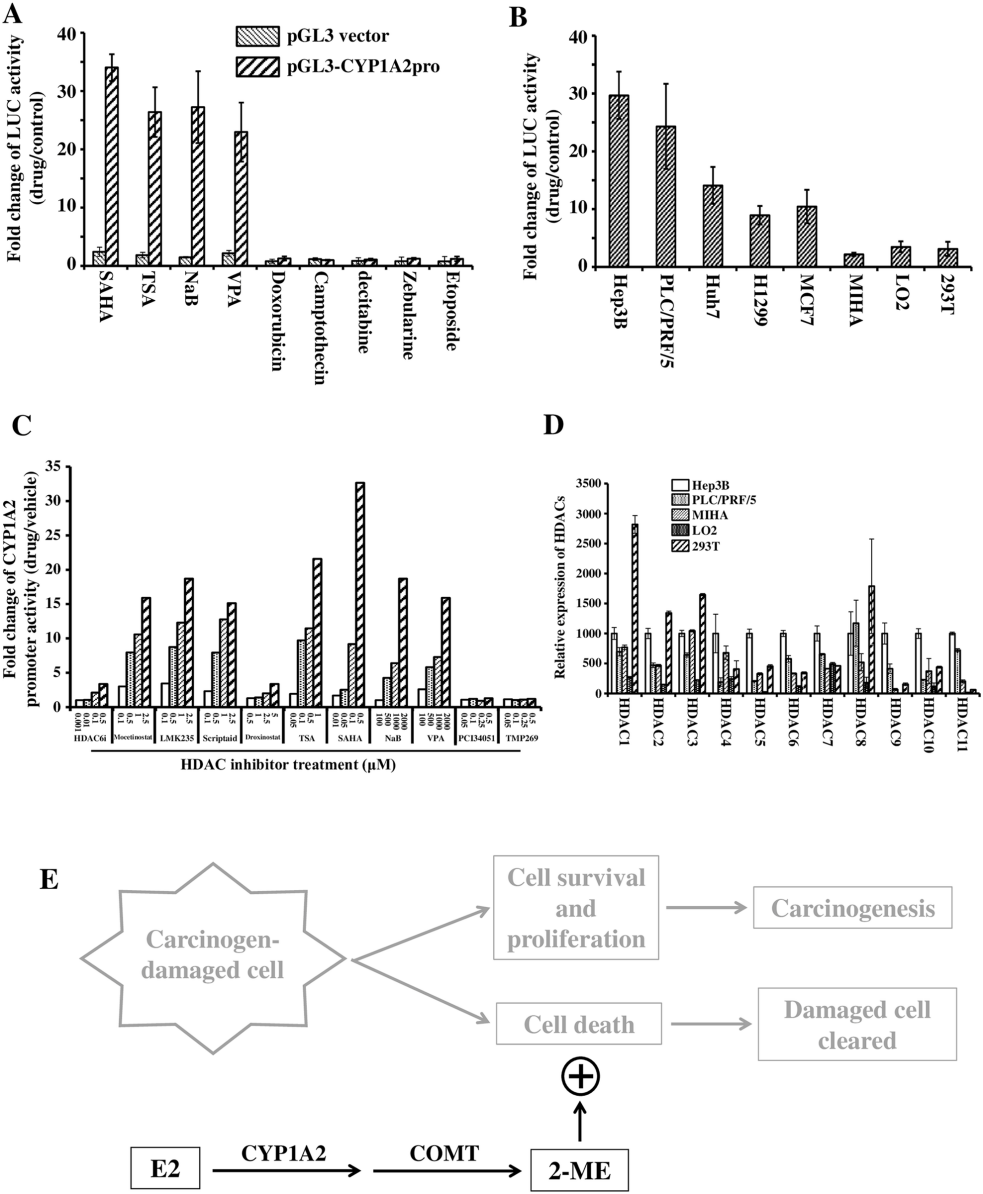
The expression of *CYP1A2* was regulated by HDAC inhibitors. **(A)** *CYP1A2* promoter is activated by HDAC inhibitors. Hep3B cells were transfected with luciferase reporter plasmids and then treated for 24 hours with the drugs of the following concentrations before being harvested for Luc assays: 2μM SAHA, 1μM TSA, 5mM sodium butyrate (NaB), 5mM VPA, 0.2μg/ml doxorubicin, 2μM camptothecin, 5μM decitabine, 5μM zebularine, and 10μM etoposide. The concentrations were determined in preliminary cell proliferation assays in which the drugs caused obvious changes in cell growth. The data are expressed as fold changes of luc reading relative to that of vehicle-treated cells of same transfection (control). Each column represents the mean ± s.d. of three independent experiments. **(B)** Inductive effect of HDAC inhibitor on *CYP1A2* promoter is higher in cancer cells. Tumorigenic (Hep3B, PCL/PRF/5, Huh7, H1299, MCF7) or non-tumorigenic (MIHA, LO2, 293T) cells were transfected with *CYP1A2* promoter-luciferase reporter plasmids and treated with 2μM SAHA for 24 hours before being harvested for Luc assays. The data are expressed as fold changes of luc reading relative to that of vehicle-treated cells (control). Each column represents the mean ± s.d. of three independent experiments. **(C)** Different HDAC inhibitors exert different effects on *CYP1A2* promoter activity. The data are expressed as fold changes of luc reading relative to that of vehicle-treated cells (control). Concentrations of the drugs were determined by referring to the reported Half Maximal Inhibitory Concentrations (IC50) of the inhibitors against various HDAC proteins. **(D)** Expression levels of “classical” *HDAC* genes in HCC cells and non-tumorigenic cells. The expression levels were normalized with β-Actin mRNA levels. Each column represents the mean ± s.d. of four technical replicates. **(E)** Hypothesis on how E2 contributes to the clearance of carcinogen-damaged cells and consequently suppresses carcinogenesis.

The mechanism underlying the suppression of CYP1A2 expression in HCC was also investigated. The 5kb promoter region upstream of the *CYP1A2* TSS was cloned in a luciferase (Luc) reporter vector. It was found that the promoter activity could be greatly induced by all the four HDAC inhibitors in the experiment, suberoylanilide hydroxamic acid (SAHA), trichostatin A (TSA), sodium butyrate (NaB), Valproic acid (VPA), but not by inhibitors against other chromatin modifying enzymes, such as doxorubicin, camptothecin, etoposide (Topoisomerase inhibitors), decitabine (5-aza-2'-deoxycytidine) and zebularine (DNA methyltransferase inhibitors) “Fig 5A”. More interestingly, the induction effect of HDAC inhibitors on *CYP1A2* promoter was much more robust in cancer cells (Hep3B, PCL/PRF/5, Huh7, H1299, MCF7) than that in non-tumorigenic cell lines (MIHA, LO2, 293T) and the strongest induction was observed in HCC cells Hep3B, PCL/PRF/5 and Huh7 “Fig 5B”.

Referring to the reported Half Maximal Inhibitory Concentrations (IC50), we have also performed the dose-response analysis to examine the effects of various HDAC inhibitors, which possess different efficacies against particular HDACs, on *CYP1A2* promoter activity and found that not all of the HDACs were involved in regulating *CYP1A2* promoter activity. For example, PCI-34051, a specific HDAC8 inhibitor [29], showed no effect on promoter activity even at very high concentration “Fig 5C”, indicating HDAC8 may not contribute to the regulation of *CYP1A2* expression. Some of the tested inhibitors, such as SAHA and TSA, only work against “classical” HDACs [30,31]. Therefore we examined the expression levels of these *HDAC*s in several cell lines and found expression levels of some *HDAC*s, such as *HDAC9* and *HDAC11*, were high in HCC cells compared with that in non-tumorigenic cells “Fig 5D”.

## Discussion

Increasing evidence has indicated that E2 can suppress the proliferation, growth and metastasis of HCC and prevent hepatocarcinogenesis [5,13,14,24,25,32–36]. Consistently, we also observed the inhibitory effect of E2 on HCC *in vitro* and *in vivo*. We further demonstrated that E2 could additively enhance the anti-HCC effect of sorafenib, an agent that is frequently used to treat advanced HCC. This finding may be therapeutically significant. Recent studies have shown some limits of sorafenib treatment due to low tolerance and resistance and thus it is suggested that sorafenib needs to be used in combination with other anti-tumor agents [28]. Accordingly, E2 appears to be one of such potential agents that may be administered together with sorafenib.

In this study, we observed that *ERα* expression was significantly repressed in HCC, which was consistent with previous report [37]. The downregulated *ERα* expression in HCC suggested that the inhibitory effect of E2 may be achieved via stimulating ERα. However, several pieces of evidence obtained in our work do not support this concept. First, the overexpression of ERα did not influence the inhibitory effect of E2. Second, the ERα antagonist fulvestrant did not block the E2-mediated inhibition. Finally, E2 at low concentrations could activate its nuclear receptors ERs (S1 Table) but was unable to inhibit HCC. The inhibitory effect was observed only at higher concentrations, which is similar to the dosages reported in animal models [13,25]. Although the overexpression of *GPR30* was found to be able to enhance the cytotoxic property of E2 in HCC cells, its expression was not significantly dysregulated in HCC. Thus the involvement of GPR30 in E2-mediated inhibition of HCC is likely to be minimal. The inhibitory effect of E2 is also unlikely to be associated with ERβ since its expression was not changed in HCC and its overexpression did not affect the E2-mediated inhibition in HCC. Our results have also suggested that wild-type p53 (wt P53) is also unlikely to play a role in the E2-mediated inhibition in HCC since E2 shows similar inhibitory effects in HCC cells with and without wt P53. Whether the E2-mediated inhibition in HCC is related to P53 mutants has not been tested in this study and thus remains an interesting question.

The metabolism of E2 mainly takes place in the liver and E2 metabolites may exert biological effects on the target cells [38]. Among various E2 metabolites, 2-ME has received increasing attention due to its potent anti-tumor property [39]. Interestingly, as a major E2 metabolite, 2-ME has lost the estrogenic activity but gained a significant ability to inhibit cancer cells [40]. In HCC, a number of publications have demonstrated that 2-ME could inhibit HCC via multi-channels including suppression of angiogenesis, cell cycle arrest, and induction of apoptosis [20,21]. Unfortunately, there are very limit studies to examine CYP1A2, CYP3A4 and COMT, three key enzymes responsible for the production of 2-ME [18,19], in HCC. In this study, we have shown that the expression levels of *CYP1A2*, *CYP3A4* and *COMT* were all reduced in HCC. The observation of low *CYP1A2* expression in HCC was also reported by another group recently [41]. However, among these three enzymes, only CYP1A2 but not CYP3A4 and COMT enhanced the inhibitory effect of E2. The fact that suppression of *COMT* expression may reduce the effect of *CYP1A2* overexpression also indicates CYP1A2/COMT metabolic pathway plays an important role in mediating the inhibitory effect of E2 “Fig 3E”. These findings have therefore indicated a positive role of CYP1A2 in E2-induced inhibition of HCC. Indeed, the E2 turnover rate was markedly increased by CYP1A2, as the overexpression of *CYP1A2* significantly reduced the level of E2 but elevated the level of E2 metabolite 2-ME which possesses a well-known anti-HCC property [20,42–44]. Our results have for the first time indicated that CYP1A2 may mediate the inhibitory effect of E2 by facilitating E2 metabolism to generate the cytotoxic 2-ME in HCC and that the reduction of *CYP1A2* expression observed in HCC may thus damage the metabolic homeostasis of liver to fight against cancer, which may lead to the development and progression of HCC.

We have noted that different liver cancer cell lines responded differently to E2 treatment, hence we examined the expression levels of *CYP1A2* and *COMT* in these cell lines but didn’t get meaningful results to explain the phenomena. In addition, the expression of *CYP1A2* is extremely low in all of the cell lines, thus the above mentioned difference should not be caused by CYP1A2. It is likely that factors besides CYP1A2/COMT may also mediate the inhibitory functions of E2. Further experiments need to be done in order to fully understand the mechanism underlying E2-mediated inhibition. It was also noted that the effective concentration of E2 used in the work was much higher than the reported physiological level of the circulation system [45]. There are several possible explanations for this phenomenon. First, the structure or activity of the synthetic E2 may be different from the endogenous hormone molecule. This may explain why a high dose of E2 is usually applied in clinical therapies and animal works [46]. Second, although 10–30μM E2 was applied in 48-hour inhibitory experiments. However, 5μM E2 could already completely prevent HCC cells from growing in a 10-day culture. Furthermore, 1μM E2 was enough to result in a slowdown in migration of HCC cells. These results indicated that the lower concentration of E2 was still effective in suppressing cancer cells in the long-term treatment. Third, it has been reported that concentration of E2 in the blood did not directly represent the concentration in the organs [47]. Since liver is the main organ for E2 metabolism, the actual E2 concentration in liver is possibly higher than that in the circulation system. Nevertheless, further investigations in this issue are needed.

We have also preliminarily explored the underlying mechanism for the reduction of *CYP1A2* expression in HCC. We found that *CYP1A2* promoter activity was greatly induced by HDAC inhibitors in various cancer cells, especially in liver cancer cells. These findings suggest that the repression of *CYP1A2* expression is possibly due to the deregulated HDAC activity which is always observed in cancer cells [48]. The highest induction observed in liver cancer cells is also consistent with the specificity of *CYP1A2* expression in hepatocytes [49]. We have also found that not all of the HDAC proteins are involved in regulating CYP1A2 expression in HCC cells. By analysing the results of luciferase assay and RT-qPCR, we may figure out potential HDACs that may play key roles in HCC cells for further investigation. Moreover, it will be very interesting to further investigate how the HDACs may mediate the regulation on *CYP1A2* expression in cancer cells, especially in liver cancer cells. In addition, induction of *CYP1A2* promoter activity indicated that the repression of *CYP1A2* expression in cancer cells is reversible, providing potential options for pharmacological interventions to correct this pathological disturbance.

In conclusion, our study provides evidence that HCC is associated with the downregulation of *CYP1A2* expression and the overexpression of *CYP1A2* enhances the inhibitory effect of E2 via facilitating E2 metabolism to generate the cytotoxic 2-ME. Considering the fact that CYP1A2 is primarily expressed in liver and as the major enzyme for E2 metabolism [18,19,50], the coincidence of CYP1A2 functions in enhancing the inhibitory effect of E2 and its extremely low expression in HCC may offer some hints for new therapeutic developments and also represent a great support to our hypothesis that E2 metabolism is involved in the gender disparity of HCC incidence. It is commonly agreed that carcinogenesis is initiated when the survival-death balance is broken in carcinogen-damaged cells. Therefore our results have suggested that E2 may contribute to the clearance of damaged cells by enhancing the cell death effect via CYP1A2/COMT metabolic pathway (Fig 5E) and consequently reduce the risk of HCC in females.

## Supporting Information

S1 Fig GPR30 activity. (PDF)

S2 Fig Migration rates of Hep3B stable cells under E2 treatment. (PDF)

S1 Table Clinical information of HCC patients. (PDF)

S2 Table Activity of estrogen receptors ERα and ERβ. (PDF)

S3 Table Activities of CYP1A2, CYP3A4 and COMT. (PDF)

S4 Table Primers used in RT-qPCR. (PDF)
