# Supplementary material for: Cytochrome P450 1A2 Metabolizes 17β-Estradiol to Suppress Hepatocellular Carcinoma
Source: PLoS One. 2016 Apr 19;11(4):e0153863. doi: 10.1371/journal.pone.0153863 (PMC4836701; doi:10.1371/journal.pone.0153863)

S1 Fig. GPR30 activity. The assay for GPR30 activity is designed based on the fact that estrogen induced apoptosis in GPR30-overexpressing cells. Hep3B cells were transfected with GPR30 expression plasmids or empty vector and grown for 24 hours. Then the cells were seeded at 5×10^3 cells/per well of 96-well plates and cultured for 24 hours. Afterwards the cells were treated with 30μM β-estradiol for 48 hours and analyzed for the proliferation by MTT. The proliferation rate is expressed as the value of treated cells versus that of the corresponding untreated cells (=100%). Each column represents mean+s.d. of data obtained from four replicate wells. * $p<0.05$.

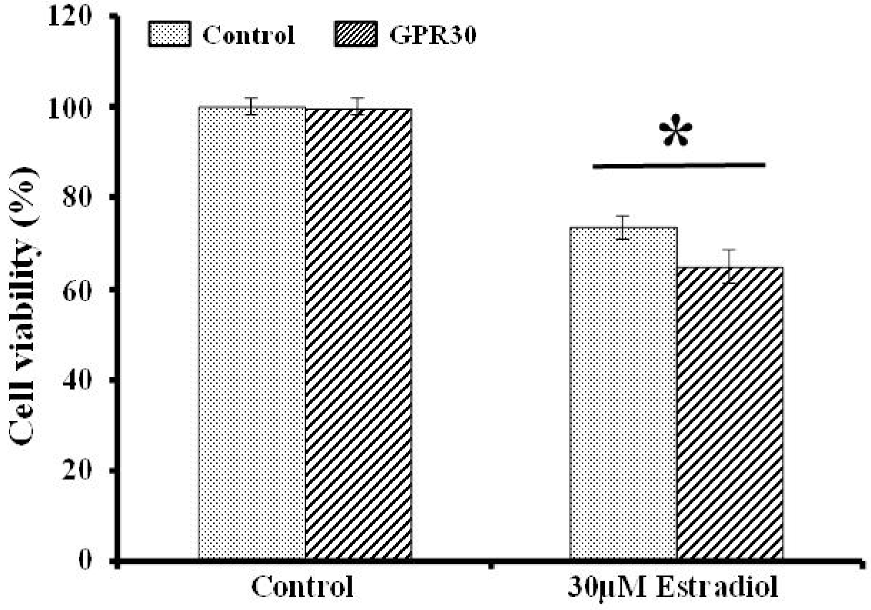

Supplement: S1 Fig — (PDF) [file pone.0153863.s001.pdf]
