# Supplementary material for: Cytochrome P450 1A2 Metabolizes 17β-Estradiol to Suppress Hepatocellular Carcinoma
Source: PLoS One. 2016 Apr 19;11(4):e0153863. doi: 10.1371/journal.pone.0153863 (PMC4836701; doi:10.1371/journal.pone.0153863)

S2 Fig. Migration rates of Hep3B stable cells under E2 treatment. Control and CYP1A2 stable Hep3B cells were grown to confluence and scratched with a rubber policeman. The cells were then grown in medium supplemented with 1μM E2 for 10 days. Microscopic images were taken at indicated time points.

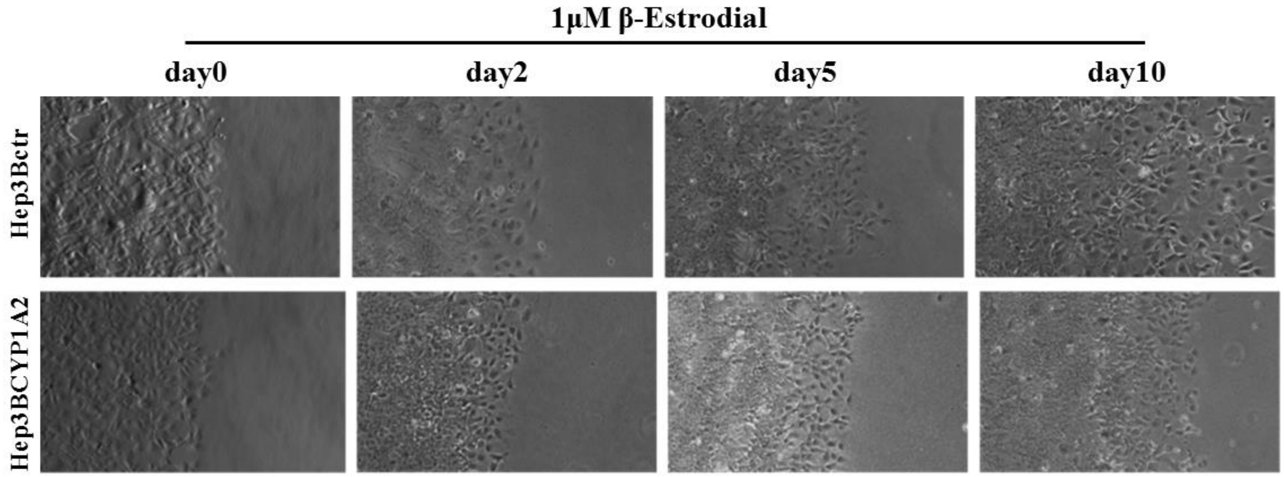

Supplement: S2 Fig — (PDF) [file pone.0153863.s002.pdf]
