# Supplementary material for: Cytochrome P450 1A2 Metabolizes 17β-Estradiol to Suppress Hepatocellular Carcinoma
Source: PLoS One. 2016 Apr 19;11(4):e0153863. doi: 10.1371/journal.pone.0153863 (PMC4836701; doi:10.1371/journal.pone.0153863)
Supplement: S1 Table — (PDF) [file pone.0153863.s003.pdf]

S1 Table. Clinical information of HCC patients.

HCC patient	gender	age of diagnosis	AJCC staging	tumor size (cm^3)	HBV(+/-)	overall survival (month)
HCC1	Female	70	2	4.5	+	44.2
HCC2	Male	57	1	4	+	11.7
HCC3	Female	67	1	2.8	+	38
HCC4	Male	68	2	2	+	21.4
HCC5	Female	55	1	3	+	123.6
HCC6	Male	65	1	4	+	161.8
HCC7	Female	67	2	3.2	+	22.4
HCC8	Male	48	1	3.5	+	0.1
HCC9	Male	67	1	2.3	+	127.3
HCC10	Female	41	1	2.5	+	158.1
HCC11	Male	50	2	3	+	11.9
HCC12	Male	54	1	2.5	+	1.1
