# Supplementary material for: Cytochrome P450 1A2 Metabolizes 17β-Estradiol to Suppress Hepatocellular Carcinoma
Source: PLoS One. 2016 Apr 19;11(4):e0153863. doi: 10.1371/journal.pone.0153863 (PMC4836701; doi:10.1371/journal.pone.0153863)
Supplement: S2 Table — (PDF) [file pone.0153863.s004.pdf]

S2 Table. Activity of estrogen receptors ERα and ERβ. p2xEREluc reporter plasmid [Renaud HJ, et al Toxicol Sci 2011;124:261-277] was cotransfected with control, ERα or ERβ expressing plasmids into Hep3B cells and the cells were grown for 24 hours. After the cells were treated with β-estradiol of the indicated concentrations for 15 hours, the cells were harvested and lysed for luciferase assay with luciferase assay system (Promega, Madison, WI). The activity of ERα or ERβ is exhibited by the increasing luminescence intensity as the estrogen concentration is elevated.

Estradiol (nM)	Control+ERE Luc read	ER-α+ERE Luc read	ER-β+ERE Luc read
0	209	603	400
10	236	968	2812
20	223	1716	2320
50	298	1460	3875
100	224	1363	3275
200	292	1202	3576
500	247	1255	3723
