# Supplementary material for: Cytochrome P450 1A2 Metabolizes 17β-Estradiol to Suppress Hepatocellular Carcinoma
Source: PLoS One. 2016 Apr 19;11(4):e0153863. doi: 10.1371/journal.pone.0153863 (PMC4836701; doi:10.1371/journal.pone.0153863)
Supplement: S3 Table — (PDF) [file pone.0153863.s005.pdf]

S3 Table. Activities of CYP1A2, CYP3A4 and COMT. The activities of estrogen metabolizing enzymes CYP1A2, CYP3A4 and COMT were examined via cell-based analysis with respective P450-Glo™ Assays (Promega, Madison, WI). (**A**) Activity assay for CYP1A2. Hep3B cells were transfected with control or CYP1A2-expressing plasmids and grown for 24 hours. Then the cells were seeded at $3x10^4$ cells/per well of 96-well plates and cultured for 24 hours before cell-based CYP1A2 assay was performed with Promega kit (product #V8421). Each test was conducted in triplicate wells. The activity of the overexpressed CYP1A2 is demonstrated by the high luminescence intensity detected in substrate-added wells. (**B**) Activity assay for CYP3A4. Hep3B cells were transfected with control or CYP3A4-expressing plasmids and cultured for 24 hours. Then the cells were seeded at $3x10^4$ cells/per well of 96-well plates and grown for 24 hours before cell-based CYP3A4 assay was performed with Promega kit (product #V9001). Each test was conducted in triplicate wells. The activity of the overexpressed CYP3A4 is demonstrated by the high luminescence intensity detected in substrate-added wells. (**C**) Activity assay for COMT enzyme. The assay was performed based on the fact that COMT may further modify the metabolism products generated by cytochrome P450 enzymes [Zhu BT, Conney AH. Carcinogenesis 1998;19:1-27]. COMT expressing plasmid was co-transfected with CYP1A2 or CYP3A4 expressing plasmids into Hep3B cells and the cells were cultured for 24 hours. Then the cells were seeded at $3x10^4$ cells/per well of 96-well plates and grown for another 24 hours before cell-based CYP1A2 or CYP3A4 assay was performed as described above. The activity of COMT was demonstrated by the reduction of luminescence intensity caused by COMT in CYP1A2 or CYP3A4 overexpressing cells.

A

	no CYP1A2 substrate added				CYP1A2 substrate added		
	well 1	well 2	well 3		well 1	well 2	well 3
Control	45	38	46		103	124	144
CYP1A2	42	38	68		6631	6048	5075

B

	no CYP3A4 substrate added				CYP3A4 substrate added		
	well 1	well 2	well 3		well 1	well 2	well 3
Control	78	68	68		77	62	134
CYP3A4	85	67	55		295	270	197

C

	no substrate added	substrate added
CYP1A2+control	120	439
CYP1A2+COMT	158	148
CYP3A4+control	127	637
CYP3A4+COMT	133	192
