# Supplementary material for: Cytochrome P450 1A2 Metabolizes 17β-Estradiol to Suppress Hepatocellular Carcinoma
Source: PLoS One. 2016 Apr 19;11(4):e0153863. doi: 10.1371/journal.pone.0153863 (PMC4836701; doi:10.1371/journal.pone.0153863)
Supplement: S4 Table — (PDF) [file pone.0153863.s006.pdf]

S4 Table. Primers used in RT-qPCR

Gene	Primer	Sequence
ER-α	Forward	5'-TCCTGGACAAGATCACAGAC-3'
	Reverse	5'-TGCAGGAGACAGAATTTGGC-3'
ER-β	Forward	5’-ATGGATATAAAAAACTCACCAT
	Reverse	5'-TCCTGCTCAATTCCAGTATG-3'
GPR30	Forward	5'-AAGTGCACCTCCAGCCTGA-3'
	Reverse	5'-AAGAACAGATGCTCCTCACA-3'
COMT	Forward	5'- ATGATGTGGACACACTGGAC -3'
	Reverse	5'- TCCAGGAACGATTGGTAGTG -3'
CYP1A2	Forward	5'- AGCACAACAAGGGACACAAC -3'
	Reverse	5'- ATGGCCAGGAAGAGGAAGAT -3'
CYP3A4	Forward	5'- TCCTTCAGAACTTCTCCTTC -3'
	Reverse	5'- AATGAGAGAGCTCAATGCATG -3'
HDAC1	Forward	5'-CTATCAAAGGACACGCCAAGTG-3'
	Reverse	5'-ACCGGGCAACGTTACGAAT-3'
HDAC2	Forward	5'-CATGGTGATGGTGTTGAAGAAG-3'
	Reverse	5'-TCATTGGAAAATTGACAGCATAGT-3'

**HDAC3**	**Forward**	**5'-TTGAGTTCTGCTCGCGTTACA-3'**
	**Reverse**	**5'-CCCAGTTAATGGCAATATCACAGAT-3'**
**HDAC4**	**Forward**	**5'-AATCTGAACCACTGCATTTCCA-3'**
	**Reverse**	**5'-GGTGGTTATAGGAGGTCGACACT-3'**
**HDAC5**	**Forward**	**5'-TTGGAGACGTGGAGTACCTTACAG-3'**
	**Reverse**	**5'-GACTAGGACCACATCAGGTGAGAAC-3'**
**HDAC6**	**Forward**	**5'-TGGCTATTGCATGTTCAACCA-3'**
	**Reverse**	**5'-GTCGAAGGTGAACTGTGTTCCT-3'**
**HDAC7**	**Forward**	**5'-CTGGCACAGCGGATGTTTG-3'**
	**Reverse**	**5'-CTGCATTGGAGGAATGAAGCT-3'**
**HDAC8**	**Forward**	**5'-TCCCGAGTATGTCAGTATATATGA-3'**
	**Reverse**	**5'-GCTTCAATCAAAGAATGCACCAT-3'**
**HDAC9**	**Forward**	**5'-ATCAGTGACTGCTGGATAGTCTC-3'**
	**Reverse**	**5'-ATAACCTAGGACTACTGGCATGG-3'**
**HDAC10**	**Forward**	**5'-GGAAGCTCCTGTACCTCTTAGAT-3'**
	**Reverse**	**5'-GTGGAGACATGGAACATGGATAG-3'**
**HDAC11**	**Forward**	**5'-TGAAGAGCTATAGGCACTGGTGT-3'**
	**Reverse**	**5'-ACTACTGCTAAGGCAGTGATCTG-3'**

**β-Actin**	**Forward**	**5'-AGGATGCAGAAGGAGATCAC-3'**
	**Reverse**	**5'-TGTAACGCAACTAAGTCATAG-3'**
